# Editorial: Sphingolipids in cardiovascular diseases: from pathogenesis to therapeutics

**DOI:** 10.3389/fcvm.2023.1330274

**Published:** 2024-01-08

**Authors:** Massimo Collino, Marco Piccoli

**Affiliations:** ^1^ Department of Neurosciences (Rita Levi Montalcini), University of Turin, Turin, Italy; ^2^ Laboratory of Stem Cells for Tissue Engineering, IRCCS Policlinico San Donato, San Donato Milanese, Italy; ^3^ Institute for Molecular and Translational Cardiology (IMTC), San Donato Milanese, Milan, Italy

**Keywords:** sphingolipid, cardiovascular disease, ceramide, S1P, molecular target

**Editorial on the Research Topic** Sphingolipids in cardiovascular diseases: from pathogenesis to therapeutics

Despite remarkable advances in medicine, cardiovascular disease remains the leading cause of death worldwide, claiming millions of lives every year (1). These multifaceted diseases, which include conditions such as atherosclerosis, hypertension and heart failure, continue to pose new challenges to both medical research and clinical science. In recent years, sphingolipids, previously considered merely structural components of the cell membrane, have emerged as key bioactive molecules in cardiovascular health, influencing cellular signaling, inflammation and lipid metabolism (2–4).

This special issue, “Sphingolipids in Cardiovascular Diseases: From Pathogenesis to Therapeutics,” published in Frontiers in Cardiovascular Medicine, presents four articles (3 reviews and 1 original article) that represent an interdisciplinary exploration of the molecular mechanisms, risk factors, and therapeutic potential of sphingolipids. These papers improve our understanding of the central role of sphingolipids in cardiovascular disease and shed light on their complex relationships with heart health, which could lead to innovative cardioprotective strategies.

With this in mind, Borodzicz-Jazdzyk et al. delve into sphingolipids' influence of sphingolipids on essential cellular processes related to cardiac muscle. In particular, they focus on ceramide and sphingosine-1-phosphate (S1P) as bioactive and dynamic molecules that directly influence the contractility of cardiac muscle cells. Specifically, S1P exerts a negative inotropic effect via two distinct mechanisms, one involving the reduction of the L-type calcium channel current and the decrease in intracellular Ca^2+^ concentration and the other involving the inactivation of the inward K^+^ current, which shortens the duration of the action potential. Conversely, ceramide promotes positive inotropic effects by increasing the sensitivity of cardiomyocytes to calcium. These two molecules are also associated with myocardial fibrosis as they exert opposing effects. While treatment of cardiac fibroblasts with dihydrosphingosine impaired (TGF-β)-mediated collagen synthesis, administration of S1P via the Rho kinase signaling cascade increased the expression of both collagen and α-smooth muscle actin.

The ability of SLs to regulate important cellular functions leads to their direct involvement in several cardiovascular diseases, as highlighted by Ya'ar Bar et al. and Borodzicz-Jazdzyk et al. Among these pathologies, SLs are also associated with coronary artery disease (CAD) and atherosclerosis in particular. Ceramide stimulates foam cell formation, induces the expression of pro-inflammatory cytokines and promotes subendothelial infiltration of OxLDL into endothelial cells, leading to endothelial dysfunction. These effects appear to be mediated by ceramide-induced ROS formation and activation of protein phosphatase 2A (PP2A), which impair nitric oxide (NO) production and bioavailability by inhibiting eNOS. In contrast, the role of sphingosine-1-phosphate (S1P) in atherosclerosis is complex. Some studies suggest that S1P promotes atherosclerosis, while others suggest that it may attenuate atherosclerosis. Indeed, the interaction of S1P with S1PR1 might exert anti-atherosclerotic effects by reducing endothelial inflammation via phosphatidylinositol 3-kinase (PI3K) and protein kinase B signaling cascade. However, S1PR1 could activate pro-inflammatory endothelial factors such as vascular cell adhesion molecule 1 (VCAM-1) and intercellular adhesion molecule (ICAM-1) and increase the level of interleukin 6 (IL-6), which would trigger the atherogenic process. Interestingly, this antithetic effect of S1P was also demonstrated when interacting with S1PR2 and S1PR3, suggesting that further research is needed to better elucidate its role in atherosclerosis.

Another common condition, especially in the elderly, is heart failure (HF), which is categorized by left ventricular ejection fraction (LVEF) (5). SLs are thought to play a role in cellular processes that contribute to both HFpEF and HFrEF. Experimental studies have shown that ceramide (Cer) levels increase while sphingosine-1-phosphate (S1P) levels decrease in the myocardium during HF. However, the specific sphingolipid profiles and metabolic processes vary depending on the type of HF. In an elegant review, Kovilakath et al. analyzed the current literature on the relationship between SLs and HF. Overall, the results support the idea that ceramide accumulation plays a role in HF and HF-related outcomes. In particular, there is a consistent inverse relationship between long-chain fatty acid ceramides (LCFA) such as Cer C16:0 and unsaturated very long-chain fatty acid ceramides (VLCFA) such as Cer C24:1 with HF. In contrast, saturated VLCFA ceramides such as Cer C24:0 and, in some studies, sphingomyelin (SM) C24:0 are positively correlated with HF. In addition, sphingolipid ratios appear to be promising prognostic markers for the prediction of adverse cardiac events, HF and HF-related mortality. In this context, SLs have also been shown to be novel biomarkers of cardiometabolic risk (6). The original article by Berkowitz et al. addressed the cross-sectional relationship between SLs and various metabolic syndrome (MetS) parameters and other atherosclerotic risk factors. Specifically, they analyzed the sphingolipidome profile of 2,063 subjects who participated in the Midlife in the United States (MIDUS) study (7) and were divided into two distinct groups, patients with and without MetS. The results showed that several classes of sphingolipids, particularly ceramides and dihydroceramides, were positively associated with MetS and its components, while hexosylceramides and lactosylceramides were negatively associated. However, the simple β-glycosphingolipids showed a positive correlation with inflammatory and cardiovascular risk markers in individuals with MetS. Thus, sphingolipid network patterns differed according to MetS and inflammatory status, which has implications for understanding the underlying mechanisms associated with metabolic syndrome and inflammation.

In summary, the contributions compiled in this special issue provide a comprehensive overview of the intricate interactions between sphingolipids and cardiovascular diseases, ranging from metabolic dysfunction to atherosclerosis, heart failure and cardiovascular risk biomarkers. We invite readers to find these articles not only interesting but also instructive and to use them as a stimulus for future research. Understanding the role of sphingolipids in cardiovascular disorders and exploring the pharmacologic manipulation of sphingolipid pathways holds promise for the development of innovative treatments and prevention of these prevalent diseases and offers the opportunity to improve the quality of life of these patients worldwide.
